# Malignant PEComa: a case report with emphasis on clinical and morphological criteria

**DOI:** 10.1186/1471-2482-11-3

**Published:** 2011-01-27

**Authors:** Federico Selvaggi, Domenico Risio, Roberta Claudi, Roberta Cianci, Domenico Angelucci, Daniela Pulcini, Alberto D'Aulerio, Margherita Legnini, Roberto Cotellese, Paolo Innocenti

**Affiliations:** 1 Unit of General and Laparoscopic Surgery, Surgical Sciences Department, "G. d'Annunzio" University, Chieti, Italy; 2 Unit of Pathology, "G. d'Annunzio" University, Chieti, Italy; 3 Unit of Radiology, "G. d'Annunzio" University, Chieti, Italy

## Abstract

**Background:**

Malignant perivascular epitheliod cell tumor (PEComa) is a very rare entity composed of distinctive perivascular epitheliod cells with variable immunoreactivity for melanocytic and muscle markers. At present this neoplasm does not have a known normal cellular counterpart and the natural history is often unpredictable. Up to now, few cases of PEComa have been described and treatment modalities are still controversial, particularly in advanced conditions.

**Case presentation:**

We handled the case of a 42-year-old man with unresectable PEComa of the abdomen. A 7 cm hepatic hypodense lesion between segment V and VIII of the liver and diffuse intraperitoneal nodules of 0,3-3,5 cm along the right subcapsular hepatic region, were documented by a CT scan. Radiological images showed abnormal lymph nodes of the right internal mammary chain and anterior mediastinum. The patient underwent an explorative laparotomy for uncontrolled intrabdominal hemorrhage without a well-defined preoperative tumor diagnosis. At surgery, multiple lobulated nodules containing hemorrhagic fluid on the liver surface, peritoneum and omentum were confirmed. The procedure had a palliative intent and consisted of hemostasis, hematomas evacuation and omentectomy. The diagnosis of PEComa was made after surgery on the basis of morphological and immunohystochemical criteria. Radiological and intra operative findings suggest that the mass has an hepatic origin with diffuse involvement of hepatic capsule and suspensory ligaments. The patient received medical support care with blood and plasma transfusions. In our experience, PEComa was clinically malignant, leading to a fatal outcome 25 days after hospital admission of patient.

**Conclusions:**

Here we report and discuss the peculiar clinical, radiological and morphological presentation of unresectable PEComa. Although in the majority of the reported series, PEComas show a more better prognosis, our case presents with a particular aggressive biological behaviour. The importance of a correct preoperative diagnosis, the need for more effective targeted therapies based on tumor molecular knowledge and evidence-based clinical studies are emphasized together with a revision of the concerning scientific literature.

## Background

PEComa is a mesenchimal neoplasm, predominantly affecting young adults and female individuals [1]. It includes clear cell "sugar" tumor of the lung and extrapulmunary sites, angiomyolipoma, clear cell myomelanocytic tumor of the falciform ligament/ligamentum teres and rare lymphangioleiomyomatosis-like tumors [2]. PEComa has been identified at multiple anatomic sites, such as the liver, uterus, vulva, rectum, heart, breast, urinary bladder, abdominal wall and pancreas, and has been associated with few, if any symptoms, though abdominal pain and bleeding have been reported [1-4]. Preoperative differential diagnosis includes gastrointestinal stromal tumors, melanoma, clear cell sarcoma, leiomyosarcoma [5]. A well-defined preoperative diagnosis is hard to make because of non-specific radiological features [1]. Preoperative biopsy might overcome this limitation, but the data coming from current clinical practices suggest that PEComa diagnosis is usually confirmed after surgery [1,3]. The biological behaviour of PEComa varied in different cases, some of which developed metastasis, local recurrence or death [6]. We report our first case of malignant PEComa discovered in a young man who underwent an exploratory laparotomy for uncontrolled intrabdominal hemorrhage.

## Case presentation

**Figure 1:**
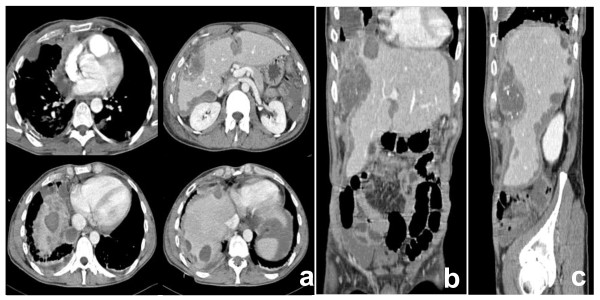
**CT appearance of PEComa: axial (a), coronal (b) and sagittal (c) reconstructions**. CT of the abdomen showed a 7 cm lesion in the right lobe of the liver with multiple ovular lesions of 0.5-3.5 cm along right triangular ligament, hypoattenuating relative to the surrounding hepatic tissue with minute calcifications. Hypodense lesions along right triangular ligament and capsular profile of falciform ligament were additionally documented. In the thorax CT images, abnormal lymph nodes of 2,8 cm in length were observed along internal right mammary chain, anterior mediastinum and aorta-lung interface. Multiple nodular lesions of the lung were documented in the right hemi-thorax.

**Figure 2:**
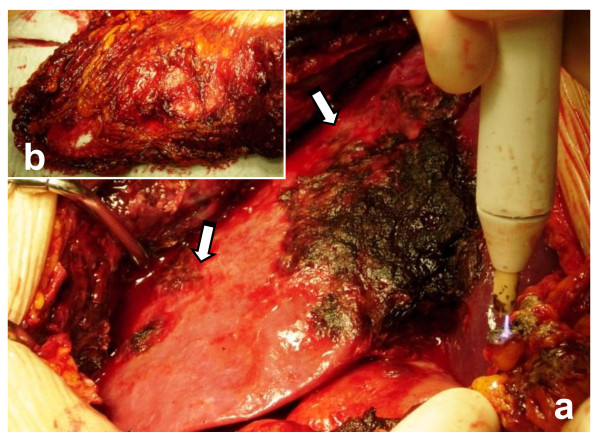
**Intraoperative appearance of PEComa**. Multiple lobulated nodules containing hemorrhagic fluid on the hepatic capsule, peritoneum surface (a) and omentum (b) associated with hemoperitoneum were observed. The surgical procedure consisted in toilette of peritoneal cavity, omentectomy and hemostasis. In our case, it was impossible to perform an oncological curative surgery.

A 42-year-old male was admitted to our observation with dyspnea, temperature, abdominal discomfort and weight loss. He had previously experienced tuberculosis infection, and his past surgical history was uneventful. The physical examination confirmed epigastric and mesogastric pain. The radiological images demonstrated right fluid pleurical collection with passive collapse of the lung inferior lobe, abnormal lymph nodes of the right internal mammary chain and anterior mediastinum (Figure 1). At the patient admission, we had drained the right symptomatic hemothorax and sent to our pathologists the pleurical fluid for cytology exam. The patient underwent an abdominal CT scan that demonstrated a 7 cm hypodense lesion between segment V and VIII of the liver with minute calcifications, inhomogeneously hypoattenuating relative to the surrounding liver parenchyma. This lesion was surrounded by a very thin capsule showing irregular limits in the cranial portion. The right hepatic lobe showed irregular profile with the presence of diffuse lesions of 0,5-3,5 cm in length, reported along right triangular ligament and subcapsular hepatic region, suspected for hematomas. Hemoperitoneum was also documented (Figure 1). According to the severe clinical condition and the hemodynamic instability, the patient underwent hepatic angiography with selective catheterization of the common hepatic artery, its branches and right diaphragmatic artery. The diagnostic phase of angiography did not document any active bleeding, as well as during the selective coaxial study of the intrahepatic arterial branches of right hemi-system and right diaphragmatic artery. An exploratory laparotomy was finally proposed. At surgery, the hepatic mass, the multiple subcapsular hematomas, and the hemoperitoneum were confirmed (Figure 2). The liver had no signs of cirrhosis. The extemporaneous biopsy showed signs of undifferentiated carcinoma. The surgical procedure consisted in omentectomy, toilette and hemostasis of the peritoneal cavity.

The histological diagnosis of malignant PEComa is based on the published criteria by Folpe and co-workers. In our case, the tumor is characterized by the proliferation of epitheliod and spindle cells. The neoplastic cells are arranged in small-nests or sheet-like patterns, traversed by a delicate vasculature consisting of a rich network of sinusoid-type blood vessels. The tumor cells showed a round to oval nucleus, often with prominent nucleolus and exhibited high nuclear pleomorphism. The tumor is comprised of a population of large polyclonal cells with abundant cytoplasm. The mitotic index is elevated (> 40 figures per 50 high power fields, HPF) and the coagulative necrosis, a prominent feature, appeared as multiple foci of variable sizes. Microscopically the tumor border was infiltrative.

**Figure 3:**
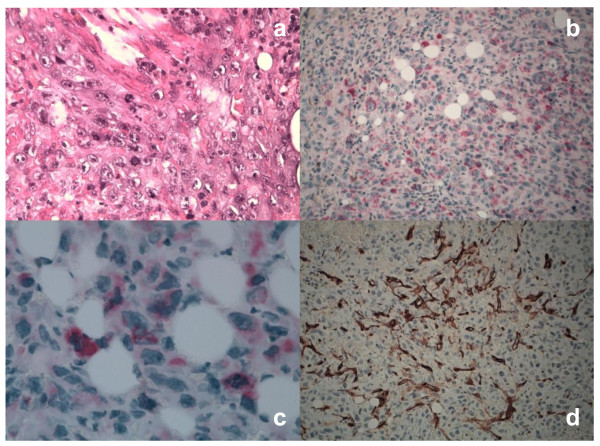
**Histological and immunohistochemical profile of PECs**. Tumor cells showed severe atypia, cytological pleomorphism, high mitotic activity and atypical mitotic figures (Hematoxylin-Eosin, x40)(a). Both epithelioid and spindle cells were diffusely positive for HMB-45 (x200)(b) and Melan-A (x63)(c) antigens in the cytoplasm. The prominent capillary network, but not tumor cells, showed CD34 positive staining (x40)(d).

**Figure 4:**
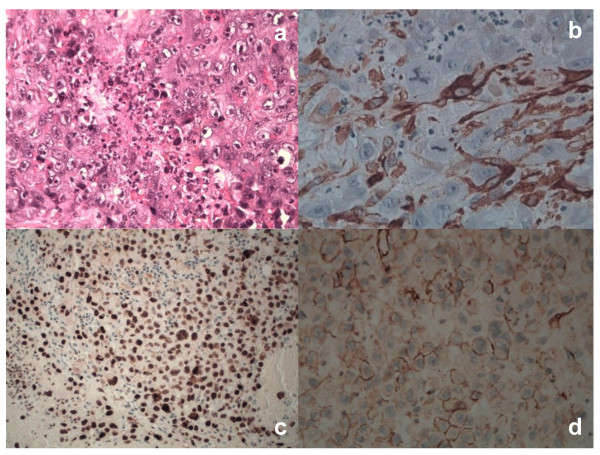
**Histological and immunohistochemical profile of PECs**. Tumor cells showed marked coagulative necrosis (Hematoxylin-Eosin, x40) (a). In PECs, SMA had a focally cytoplasmic positivity (x40)(b). Nuclear MIB-1 reactivity was documented in PECs (x200)(c). We observed a strong and diffuse membrane reactivity for CD31 in tumor cells (x40)(d). The tumor was negative for all other markers mentioned, including S-100, CKAE1/AE3, CK5, CD30.

For immunohistochemistry on formalin-fixed paraffin-embedded samples, sections were treated with H_2_O_2_/3% for 5 minutes to inhibit endogenous peroxidase and then washed in H_2_O. Antigen was unmasked by treatment with EDTA at pH 9, or with citrate buffer at pH 6 in a microwave oven (two 5-minutes courses). The slices were then held for 20 minutes at room temperature. After washing in PBS/Tween-20, sections were incubated for 30 minutes with the primary antibodies. Then, they were washed and stained with Bond™ Polymer Refine/HRP Detection Kit according to the manufacturer's protocol (Leica, Wetzlar, Germany) or Bond™ Polymer Refine Red Detection Kit (Leica) for HMB-45 an Melan-A. For negative controls, we substituted non-immune sera for the primary antibodies. The immunohistochemistry analysis demonstrated positive staining for Vimentin (Novocastra), Melan-A (Dako), HMB-45 (Dako), smooth muscle actin (SMA) (Dako), MIB-1(Novocastra), and CD31 (Novocastra). It was negative for S-100 protein (Dako), Cytokeratin-AE1/AE3 (CKAE1/AE3) (Novocastra), Cytokeratin-5 (CK5) (Novocastra), CD30 (Dako). Based on this specific immunophenotype profile, diagnosis of PEComa was made (Figure 3-4). The multiple lesions, suspected to be lymphatic metastasis in the thorax CT images, were not confirmed by a cytology exam of pleurical fluid. Infact, the cytology analysis has documented the presence of leucocytes, red globular cells and rare mesothelial cells, but not tumor cells. It was impossible in our case to confirm histologically the suspected metastatic lesions observed along lymphatic internal right mammary chain, anterior mediastinum, aorta-lung interface and of the lung.

The immediate post-operative course was uneventful and the patient received supporting therapy with blood and plasma transfusions. PEComa was clinically malignant, leading to a fatal outcome in our experience.

## Discussion and Conclusions

In 1992, Bonetti proposed the concept of perivascular ephiteliod cells (PECs) and the term PEComa was first introduced by Zamboni four years later [3]. Only in 2003, after the initial scepticism, the World Health Organization defined PEComas as mesenchymal tumors [7]. One current hypothesis is that the neoplasm derives from undifferentiated cells of the neural crest with smooth muscle and melanocytic phenotype. A second hypothesis is that PEC has a myoblastic, smooth muscle origin. A third concerns the perycitic origin [4]. PEComas have been described in different organs and are considered ubiquitous tumors. Clinical presentation is not specific and a correct pre-operative diagnosis is difficult to make. It might be often misdiagnosed with hepatocellular carcinoma, hemangioma, focal nodular hyperplasia and GIST tumor [5]. CT scan is uninformative regarding the tumor nature. Histology with immunohistochemistry techniques are needed to make the correct diagnosis [5-7]. Usually, the diagnosis of PEComa is confirmed after surgery, as reported by the current clinical practice [1,3]. About 100 PEComas has been reported, of which 38 involved the uterus [8]. Recently, Folpe and colleagues have suggested criteria for malignancy, including a size greater than 5 cm, mitotic count of more than 1 per 50 high-power fields and necrosis [7]. These criteria were observed in our clinical case. PEComa showed hypercellularity, hyperchromasia, high mitotic activity, atypical mitotic figures and coagulative necrosis. We have documented a strong and diffuse reactivity for melanocytic markers, such as HMB-45 and Melan-A, in the cytoplasm of tumor cells together with a focal positivity for SMA. In addition, we have documented a CD34 positive staining of the capillary network surrounding the tumor cells. Depending on specific microenvironment locations, PECs can modulate their morphology and immunophenotype. In some conditions, PECs can pronounce muscle features and in others they can exhibit more epithelioid morphology with strong positivity for HMB45 and weak or focal expression for SMA, as in our condition. On the basis of these peculiarities, the diagnosis of malignant PEComa was made. Interestingly, we have documented CD31 positivity in the cellular membrane of PECs. This result is not so unusual, because other reports have documented the reactivity of CD31 marker in a subset of malignant PEComa, termed PEComas not otherwise-specified [8]. PEComa of the liver is extremely unusual and only a few cases have been reported to date. To the best of our knowledge, there are less than 10 reported cases of liver PEComas [9]. The right lobe of the liver is the most common site and all cases occurred adjacent to the ligamentum teres and falciform ligament [1]. Surgical resection represents the only curative approach for primary PEComa at presentation as well as for local recurrences and metastasis, as chemotherapy and radiotherapy have not demonstrated significant benefits [8,10]. Only recently limited clinical studies have reported encouraging results in terms of therapeutic response after oral administration of mTOR inhibitor in patients with metastatic PEComa [11]. Our case represents the clinical condition of malignant PEComa of the liver presented with intrabdominal metastases. Radiological pattern and intraoperative founding suggest that the mass has an hepatic origin with diffuse involvement of hepatic capsule and suspensory ligaments. Although the majority of clinical reports on PEComa shows a better outcome, our case highlights the aggressive biological nature of a malignant subset of PEComa characterized by an infaust evolution.

## Competing interests

The authors declare that they have no competing interests.

## Authors' contributions

FS and DR analyzed the data and wrote the manuscript. DA and RC carried out the histological and bio-molecular studies. RC participated in the acquisition and interpretation of radiological data. DP, AD, RC and ML have been involved in the acquisition of clinical data and in the reviewing the scientific literature. FS and PI contributed to the final version and carried out the clinical case report. All authors read and approved the final manuscript.

## Pre-publication history

The pre-publication history for this paper can be accessed here:

http://www.biomedcentral.com/1471-2482/11/3/prepub
